# Acute Effect of Physical Exercise on Negative Affect in Borderline Personality Disorder: A Pilot Study

**DOI:** 10.32872/cpe.7495

**Published:** 2022-06-30

**Authors:** Samuel St-Amour, Lionel Cailhol, Anthony C. Ruocco, Paquito Bernard

**Affiliations:** 1 Department of Physical Activity Sciences, Université du Québec à Montréal, Montreal, Quebec, Canada; 2 Mental Health University Institute of Montreal Research Center, Montreal, Quebec, Canada; 3 Department of psychiatry and addictology, Medicine Faculty, University of Montreal, Montreal, Quebec, Canada; 4 Department of Psychology (Scarborough), University of Toronto, Toronto, Ontario, Canada; Philipps-University of Marburg, Marburg, Germany

**Keywords:** physical activity, emotion regulation, affect, emotion dysregulation, emotion induction

## Abstract

**Background:**

Physical exercise is an evidence-based treatment to reduce symptoms and negative affect in several psychiatric disorders, including depressive, anxiety, and psychotic disorders. However, the effect of physical exercise on negative affect in patients with borderline personality disorder (BPD) has not yet been investigated. In this pilot study, we tested the safety, acceptability, and potential acute effects on negative affect of a single session of aerobic physical exercise in adults with BPD.

**Method:**

After completing a negative mood induction procedure, 28 adults with BPD were randomly assigned to a 20-minute single session of stationary bicycle or a control condition (emotionally neutral video).

**Results:**

No adverse effects attributed to the physical exercise were reported and it was considered acceptable to patients. Following the negative mood induction, both conditions decreased the level of negative affect with a medium effect size but there was no significant difference between them.

**Conclusion:**

The results suggest that a single 20-minute session of physical exercise does not produce a reduction of negative affect in BPD. Future research should consider the duration and intensities of physical exercise with the greatest potential to reduce negative affect both acutely and in a more prolonged manner in this patient group.

## Highlights

- Adults with BPD have potential to benefit physical exercise.
- In this pilot study, no adverse effects were attributed to physical exercise.
- Physical exercise was as effective as a neutral video in decreasing negative affect.

Borderline personality disorder (BPD) is characterized by an instability of self-image, goals, interpersonal relationships, and affect (Gunderson et al., 2018). The one-year and lifetime prevalence rates of the diagnosis in the general population are estimated at 1.6% and 5.9%, respectively (American Psychiatric Association, 2013). Among pathogenesis models of BPD (D’Agostino et al., 2018), the biosocial developmental model proposes that emotion dysregulation is the core of BPD and underlies many characteristic behaviors (Crowell et al., 2009). This model is based on three main components: heightened sensitivity to emotional stimuli, intense reactions to emotional stimuli, and a delayed return to an emotional baseline (Crowell et al., 2009; Linehan, 1993). Difficulties regulating emotions in BPD are linked to maladaptive behaviors, which presumably function to reduce negative affect (Daros, Guevara, et al., 2018). A higher level of emotion dysregulation has also been associated with lower quality of life and daily functioning (Gratz et al., 2016) and a poorer therapeutic relationship (Gunderson et al., 2018). Emotion dysregulation has also been identified as a mechanism in other psychopathologies such as major depression and bipolar disorder, but seems to be present at a higher level in BPD than in these disorders (Gratz et al., 2016). Moreover, little is known regarding the specific dimensions of emotion dysregulation to BPD and its development compared to those of other disorders and psychopathology in general (Gratz et al., 2016). Therefore, finding diagnosis specific interventions to improve emotion regulation and help regulate negative emotions should be among the priorities for research on BPD.

From this perspective, a single session of physical exercise (PE) could be useful to help individuals with BPD regulate their emotions in the short term. The effect of a single bout of PE on affect has been the subject of two meta-analyses synthesizing the results of more than 150 studies totaling 13,000 adults in the general population (Ekkekakis et al., 2011; Reed & Ones, 2006). These meta-analyses show that a single bout of PE significantly increases positive affect with a moderate effect size (*d* = 0.47) and that this effect is higher for individuals with a lower initial level of positive affect (*d* = 0.63). Additionally, self-selected exercise intensity is more effective in increasing positive affect than an imposed intensity. The effects were moderated by cardiovascular capacity, obesity, and exhaustion tolerance (Ekkekakis et al., 2011). Similar results but with higher effect sizes have been demonstrated in adults with generalized anxiety disorder (*d* = 1.01; Herring et al., 2019), major depressive disorder (*d* = 1.25; Meyer, Koltyn, et al., 2016) and obsessive-compulsive disorder (*d* = 0.76; Abrantes et al., 2009). Another study (Stanton et al., 2016) also measured the effect of a 20-minute PE session on core affect (valence and arousal) in individuals with anxiety, bipolar, and depressive disorders and reported an increase in arousal for individuals with depressive and bipolar disorders, and an increase in valence (more positive affect) across all participants.

When studying the impact of PE on affect (Bernstein & McNally, 2017a, 2017b, 2018), researchers often experimentally induce an emotion to produce similar levels of affect across participants before exercising, or to modify affect after exercising (Barrett et al., 2007; Barrett & Bliss‐Moreau, 2009; Kuppens et al., 2013; Posner et al., 2005). Different strategies are used to induce negative emotions, including frustrating tasks (Gratz et al., 2006; Sauer & Baer, 2012), electric shocks (Seibert-Hatalsky & Wilson, 2011), videos of sexual abuse or domestic violence (Chapman et al., 2010; Daros, Williams, et al., 2018; Elices et al., 2012; Jacob et al., 2011), remembering negative memories (Sauer & Baer, 2012), music (Diedrich et al., 2016) or emotionally charged images (Sloan et al., 2010). Of these approaches, presenting videos that induce negative emotions has been shown to be the easiest, most acceptable, and most frequently used strategy (for a review, see Gilet, 2008).

To our knowledge and according to two recent reviews (Hall et al., 2019; Mehren et al., 2020; St-Amour et al., 2021), no study has yet examined the acute effects of PE on negative affect in BPD. In the present pilot study, our goal was to assess the acceptability and safety of a single session of 20 minutes of PE and quantify the effect size of the impact of such an intervention on core affect (valence and arousal) in patients with BPD following a negative emotion induction, compared to a control condition. We hypothesized that the PE session would be well accepted by the participants and that no adverse effects would be attributed by the participants to the PE condition. Based on the research conducted on participants drawn from the general population and those with psychiatric disorders, we additionally hypothesized that the PE condition would increase the valence and decrease the arousal of their core affect with a moderate effect size after the negative emotion induction procedure.

## Method

### Participants

Patients from the Relational and Personality Disorders service from the Mental Health University Institute of Montreal gave their consent to their healthcare professionals to be contacted for research. Thereafter, healthcare professionals referred patients to researchers based on their established BPD diagnosis. Researchers then contacted patients by phone and/or email and planned an appointment after a short screening of inclusion and exclusion criteria.

To be included in the study, participants were required to meet the following criteria: 18 years or older; previously diagnosed with BPD by two convergent psychological measures—Borderline Personality Questionnaire (Larivière et al., 2021) and Structured Clinical Interview for DSM-IV Axis II Disorders (BPD interview; Lobbestael et al., 2011)—by a psychiatrist from the Relational and Personality Disorders service from the Mental Health University Institute of Montreal; outpatient status at the Mental Health University Institute of Montreal; physically inactive (i.e., engaging in less than 150 minutes of physical activity weekly as measured with the SIMple Physical Activity Questionnaire [SIMPAQ]; Rosenbaum et al., 2020); and have a sufficient written and oral comprehension of French for the completion of the study. Participants were excluded if they had an active psychotic episode, a functional limitation preventing them from using a stationary bicycle, or a severe substance use disorder other than tobacco and cannabis. Since active individuals in general population seem to better regulate their negative affects (Bernstein et al., 2019), by recruiting inactive individuals only, we isolated the acute effect of PE from its chronic effect.

All participants gave their informed consent by reading and signing a consent form. The research protocol was approved by the ethics board committee from the University Integrated Center of Health and Social Services of Montreal. Participants were given $50 CAD compensation at the end of the protocol.

### Safety and Acceptability

At the end of the PE session, the participants reported how they felt and were asked to call or write to the research assistant to report any adverse effects that may have occurred in the following days. At the end of the session, the researcher asked each participant: “How did you feel about the physical exercise you just did?” The answer to this question was written on the participant’s results sheet. The psychiatrist from the Mental Health University Institute of Montreal (co-investigator in this study) who referred the participants was asked to report any adverse effects he noticed with his patients to the rest of the research team.

### Baseline Measures

Upon completion of the consent form, participants filled out questionnaires about sociodemographic, physical activity, and mental health information. The sociodemographic questionnaire included questions on sex, age, education level, marital status, height, weight, household income, psychiatric history, and current medications. Additional measures were used to assess physical activity, depression, BPD, and substance use symptoms. The SIMPAQ is a validated five-item physical activity questionnaire for use with adults with severe mental health disorders with good reliability, although it has not been validated in adults with BPD (Rosenbaum et al., 2020). The *Beck Depression Inventory-Short Form* (BDI-SF) is a 13-item questionnaire that provides a rating of depression symptom severity (Steer et al., 1997) and has been used in adults with BPD (Hasler et al., 2014). For each item, answers are rated using a score from 0 to 3, producing a total score ranging from 0 to 39, with a score over 9 indicating a risk of moderate-to-severe depressive episode (Furlanetto et al., 2005). This questionnaire has been thoroughly validated in adults with psychiatric illness with Cronbach’s α ranging from 0.83 to 0.96; however, the measure has not been validated specifically in adults with BPD (Wang & Gorenstein, 2013). The short form of the *Borderline Symptom List* (BSL-23) is a self-rating scale that assesses the severity of BPD symptoms and has been validated in adults with BPD, with a Cronbach’s α of 0.94 (Nicastro et al., 2016). Each item is answered on a 5-point Likert scale ranging from 0 to 4, generating a total score ranging from 0 to 92. The questionnaire instructions were adapted in our protocol: participants self-reported their symptom severity for the day preceding the study and not the previous month (note that the validity of this form has not been tested).

Since there is a high prevalence of substance use disorder in adults with BPD (Kienast et al., 2014) and substance use is linked to less PE (Abrantes & Blevins, 2019; Lisano et al., 2018; Martens et al., 2006; Werneck et al., 2019), three questionnaires were administered to assess substance use in our sample. The *Cigarette Dependence Scale* (CDS) evaluates cigarette addiction with 5 items answered on a 5-point Likert scale from 1 to 5. A global score of at least 16 indicates addiction. This questionnaire has been validated with individuals with BPD with a Cronbach’s α of 0.89 (Etter et al., 2009). The *Cannabis Abuse Screening Test* (CAST) is a 6-item questionnaire assessing cannabis use (Legleye et al., 2007). A score of at least 3 is associated with a problematic use risk. The questionnaire has good validity (Cronbach’s α = 0.81) but has not been specifically validated in adults with BPD. The *Alcohol Use Disorder Identification Test* (AUDIT) short form (3-item) was used to assess risk for alcohol use disorder. A score of at least 3 for women and 4 for men indicates a high risk of alcohol use disorder. This questionnaire has been validated in adults with personality disorders with an estimated sensitivity of 87.1% (Dawson et al., 2005).

The *Difficulties in Emotion Regulation Scale* (DERS) is a 36-item questionnaire that was used to measure different aspects of emotion regulation difficulties. Each item is answered on a 5-point Likert scale ranging from 1 to 5, with the total score of the questionnaire ranging from 36 to 180. The DERS has been validated in individuals with BPD with a Cronbach’s α of 0.94 (Côté et al., 2013). We used four items from the *Dimensions of Openness to Emotions* (DOE-IT) questionnaire, with each item representing an emotion regulation strategy regrouped into two categories: relaxation and physical activation. For these four items, participants were asked to report how frequently they engaged in the strategy, and to what extent the strategy was effective (or how effective they think it would be) on two 5-point Likert scales from 0 to 4. The four items were: “1-Listen to music corresponding to my affective state (e.g., that soothes me when I’m anxious or wakes me when I’m asleep); 2-Let the different feelings, impressions or noises act on me without directing them; 3-Let all the impressions and sensations go as they are; 4-Get physically active, move, walk a few steps.” The original full questionnaire has been validated with adults with BPD with Cronbach’s α ranging from 0.67 to 0.83, depending on the subscales (Haymoz & Reicherts, 2015).

### Experimental Procedure

**Figure 1:**
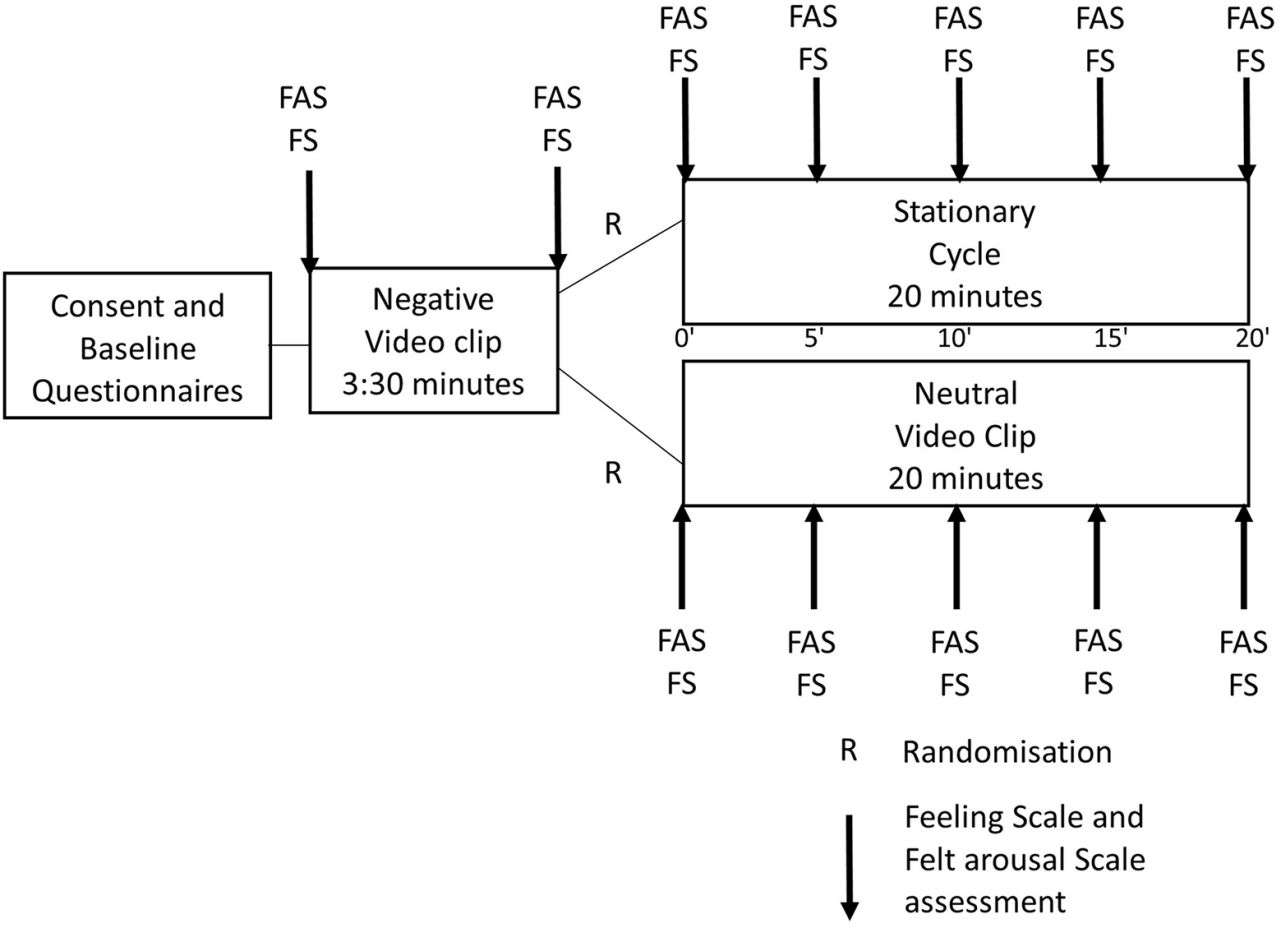
Research Protocol Schema *Note.* Negative emotion induction was presented after baseline questionnaires but before randomization to unify participants’ affects before the protocol. Time of the measurements is indicated in minutes from the beginning of the protocol between the boxes representing both groups.

Figure 1 describes the experimental procedure, including the administration of the questionnaires, negative emotion induction, and randomization to experimental conditions. Participants attended the session individually between 4p.m. and 6p.m. Participants were not instructed to refrain from using psychotropic substances (coffee, tobacco, cannabis, etc.) before the experiment. In the negative mood induction procedure, participants watched a scene lasting 3 minutes and 30 seconds from the movie *Silence of the Lambs* showing a pursuit in a dark and dirty basement. This movie clip has been shown to induce negative emotions in adults with BPD (Chapman et al., 2010; Kuo & Linehan, 2009). After the scene, participants were randomized with a heads or tails phone app to a condition, either 20 minutes of PE or an emotionally neutral video of 20 minutes (control). Fourteen participants were randomized to each condition. The PE session consisted of 20 minutes of stationary bicycle (*Life Fitness* Life Cycle 9500HR recumbent bicycle). Participants were instructed to cycle at an intensity they can maintain with pleasure for 20 minutes (Meyer, Ellingson, et al., 2016). They were also suggested the target of 11-13 on the *Borg Scale* (Borg, 1998), which was used to measure PE intensity, to help them find a low-moderate intensity in which they could be comfortable. The *Borg Scale* ranges from 6 to 20 and includes visual cues to help participants rate their PE intensity. Participants were allowed to change the load and cycling speed at will to maintain the desired intensity. There was no practice run and the participants did not receive any encouragement through the session, but they were supervised by a member of the research team in case they needed something or had a problem. The control condition consisted of the first 20 minutes of the movie *Baraka*, which has been validated to be emotionally neutral (Liu & McNally, 2017). This is a video documentary showing images of landscapes, people, and cultural rituals from around the world, with a soothing musical background and without dialogue or commentaries.

### Affect Measurement

An experimental procedure was implemented to induce a state of negative affect, which is a common approach in affective science research (Barrett et al., 2007; Barrett & Bliss‐Moreau, 2009; Kuppens et al., 2013; Posner et al., 2005). Consequently, core affect was selected as the main outcome of our study. *Core affect* refers to any mental state of pleasure or displeasure with a degree of arousal (Russell, 2003). The properties of core affect (i.e., pleasure/displeasure and arousal) are brain representations of changes in autonomic and hormonal systems of the body and regulation efforts (Barrett, 2009; Ekkekakis, 2013; Kuppens et al., 2013), and are continuously changing over time. Core affect was measured before and after the induction procedure, at the beginning of the experiment, at 5, 10 and 15 minutes into the experiment, and again at the end of each experimental condition, using two 11-point analog scales for a total of 7 measurements. The *Feeling Scale* (FS; Hardy & Rejeski, 1989) was used to measure affective valence (positive or negative). The instructions were to “estimate how good or bad you feel right now.” Anchors are provided at 0 (neutral) and odd integers, ranging from -5 (very bad) to +5 (very good). The *Felt Arousal Scale* (FAS; Svebak & Murgatroyd, 1985) was used to measure arousal. It ranges from 1 to 6 with half points. The instructions were to “estimate how aroused you feel right now” (low arousal meaning calm or fatigued and high arousal meaning anxious or energized). Anchors are provided at 1 (low arousal) and 6 (high arousal). The FS and FAS items have been used in numerous studies, including with adults who have severe psychiatric illness (Bernstein & McNally, 2017b; Edwards et al., 2018; Herring et al., 2019; LeBouthillier & Asmundson, 2015; Meyer, Ellingson, et al., 2016; Schuch et al., 2014), and are strongly correlated with the *Self-Assessment Manikin* (Unick et al., 2015).

### Statistical Analysis

Participants’ characteristics were compared between experimental conditions. Quantitative variables were compared between conditions using *t*-tests for Gaussian variables (according to the Shapiro-Wilk test) and Mann-Whitney tests otherwise. FS scores were transformed by adding 5 to produce only positive scores for the analysis. FAS scores were also transformed by multiplying them by 2 and subtracting 1 to create whole numbers only. A paired-samples *t*-test was used to examine the effects of the emotion induction. Linear mixed effect models were fitted to examine the effects of acute PE on affective valence and arousal measures. Participants were included as a random effect. All the prerequisites were met for conducting t-tests and linear mixed models. All statistical analyses were carried out with R 4.0, and the nlme and ggplot2 packages (Pinheiro & Bates, 2006). Data and analysis coding are available in open access in the Open Science Framework account of the first author (https://osf.io/ncd6r/). Post hoc achieved power analysis were carried out with G*Power 3.1.9.7 (Faul et al., 2007).

## Results

### Sample Characteristics

Twenty-eight adults (21 women) with BPD participated in the study. They were aged 19 to 56 with a mean of 36.8 (*SD* = 11.5). Sixteen participants were considered smokers (8 in each group) and 19 cannabis users (9 in the PE group and 10 in the control group). After randomization, our control group had a significantly lower household income, χ^2^(4) = 15.6, *p* = .004, and higher DERS, *t*(25) = 2.42, *d* = 0.93, *p* = .023, score than the PE group. Participant characteristics are reported in Table 1.

**Table 1:** Sample Characteristics at Baseline

Variables	PE (*n* = 14)	Control (*n* = 14)
**Age (*SD*)**	37.29 (10.79)	36.35 (12.51)
**Female (male)**	8 (5)	13 (1)
Marital Status		
Single/divorced/widow	11	12
Married	3	2
**Body mass index (*SD*)**	32.75 (10.26)	26.37 (6.83)
**Antidepressant user**	9	6
**Antipsychotic user**	6	9
**Other psychotropic user**	4	4
Education		
Elementary school	3	4
High School	2	1
Professional school	5	6
College	3	3
University	1	0
Household income*		
< 20,000$	0	7
20,000$-39,999$	11	4
40,000$-59,999	0	3
60,000$ and over	1	0
Do not know	2	0
BDI score (*SD*)	14.15 (6.91)	16.46 (5.11)
Min	1	9
Max	26	26
BSL-23 score (*SD*)	20.69 (16.26)	25.46 (17.55)
Min	5	0
Max	54	58
DERS score (*SD*)*	103.08 (29.49)	122.92 (15.54)
Min	50	97
Max	137	164
DOE-IT		
1- Listen to music		
Frequency (*SD*)	3.00 (1.18)	2.71 (1.44)
Efficiency (*SD*)	3.00 (1.04)	2.46 (1.13)
2- Let the feeling act on me		
Frequency (*SD*)	1.36 (1.45)	1.86 (1.29)
Efficiency (*SD*)	1.57 (1.40)	2.23 (0.73)
3- Let the feeling go		
Frequency (*SD*)	1.07 (1.27)	1.50 (1.35)
Efficiency (*SD*)	1.36 (1.45)	1.62 (1.26)
4- Get physically active		
Frequency (*SD*)	2.14 (1.23)	2.57 (1.34)
Efficiency (*SD*)	2.50 (1.29)	3.08 (0.76)
**CDS score/Smokers (*SD*)**	16.13 (1.25)	15.63 (1.51)
**CAST score/Cannabis users (*SD*)**	15.00 (6.61)	14.00 (6.88)
**AUDIT score (*SD*)**	6.15 (3.11)	6.00 (2.48)

*Note.* BDI = Beck Depression Inventory; BSL-23 = Borderline Symptoms List short version; DERS = Difficulties in Emotional Regulation Scale; DOE-IT = Dimension of Openness to Emotions; CDS = Cigarette Dependence Score; CAST = Cannabis Abuse Screening Test; AUDIT = Alcohol Use Disorder Identification Test.

*p < .05 when comparing both groups.

### Safety and Acceptability

An adverse effect was reported in two participants. Both participants attributed this adverse effect to the negative emotion induction procedure, which reportedly triggered psychotic symptoms (hallucinations and distress) in one participant, leading to a need for psychiatric care immediately after completion of the protocol. However, the data collected for this participant was similar to those collected for other participants. Therefore, we kept these data for analyses. It also reminded another participant of an aggression that person had reportedly experienced, which produced a drastic increase in the participant’s anxiety. It forced the participant to take a break at the 10-minute mark of the PE session and led the person to increase their alcohol consumption in the following week to a point where they sought emergency psychiatric care. Given that the participant interrupted the experiment, that individual was excluded from our analyses of the effect of the PE session. On the other hand, there were no reported adverse effects related to either the PE or control condition in the days following the protocol. All participants responded to the question, “How did you feel about the physical exercise you just did?” with positive answers (felt great, made them feel good, enjoyed exercising, etc.). However, three participants also expressed a slight discomfort related to PE (exhaustion, muscular fatigue, breathlessness).

### Mood Induction

**Figure 2:**
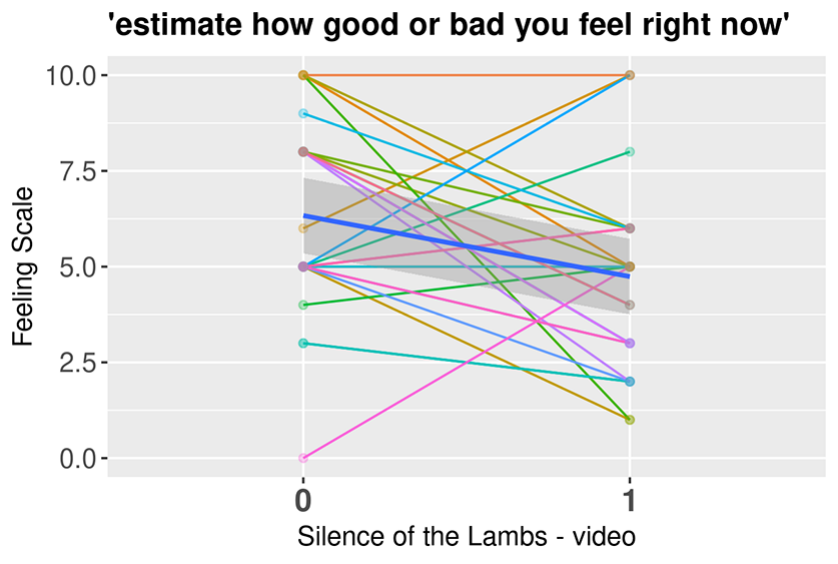
Negative Emotion Induction’s Effect on the Feeling Scale by Participants *Note.* Spaghetti plot with each line representing a participant. The bold blue line indicates the mean value of affect surrounded by a darker gray area representing the confidence interval.

**Figure 3:**
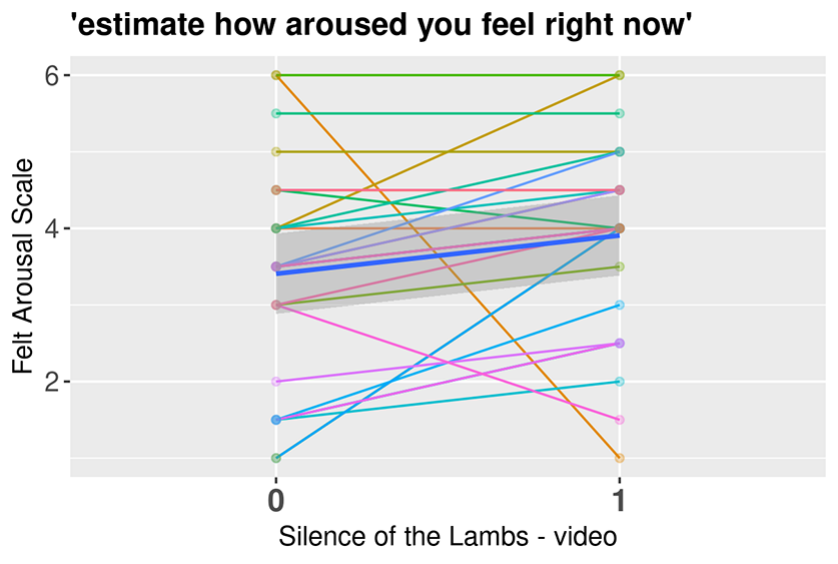
Negative Emotion Induction’s Effect on the Felt Arousal Scale by Participants *Note.* Spaghetti plot with each line representing a participant. The bold blue line indicates the mean value of arousal surrounded by a darker gray area representing the confidence interval.

The valence of affect was significantly more negative (*FS*) after (*M* = -0.36, *SD* = 2.59) the mood induction than before (*M* = 1.29, *SD* = 2.49), *t*(26) = 2.41, *p* = .023, *d* = 0.46, but the clip did not impact arousal (*FAS*), *t*(26) = -1.79, *p* = .086. However, there were individual differences in these effects: the emotion induction succeeded in increasing negative affect in 18 participants, whereas 10 participants reported no change or a decrease in negative affect. The FS and FAS data for each participant from the emotion induction are presented in Figures 2 and 3, respectively.

### Effects of PE on Negative Affect

**Figure 4:**
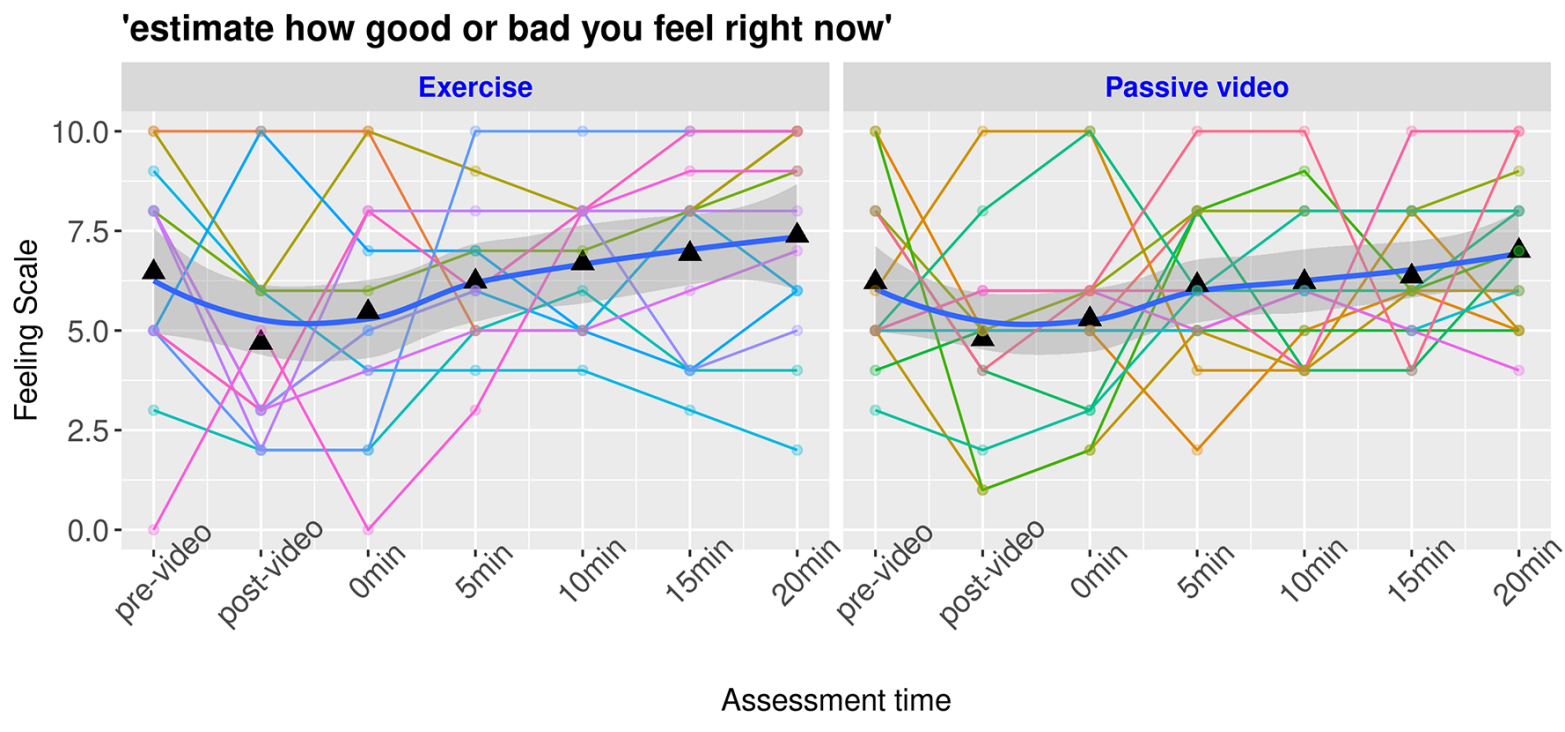
Protocol's Effect on the Feeling Scale by Participants *Note.* Spaghetti plots with each curve representing a participant with a smooth representation of the group effect with confidence intervals of the curve. The bold blue line indicates the mean value of affect valence surrounded by a darker gray area representing the confidence interval. Pre- and post video marks indicate evaluation before and after emotion induction. 0min through 20min marks indicate the time from the beginning of the condition (exercise or control).

**Figure 5:**
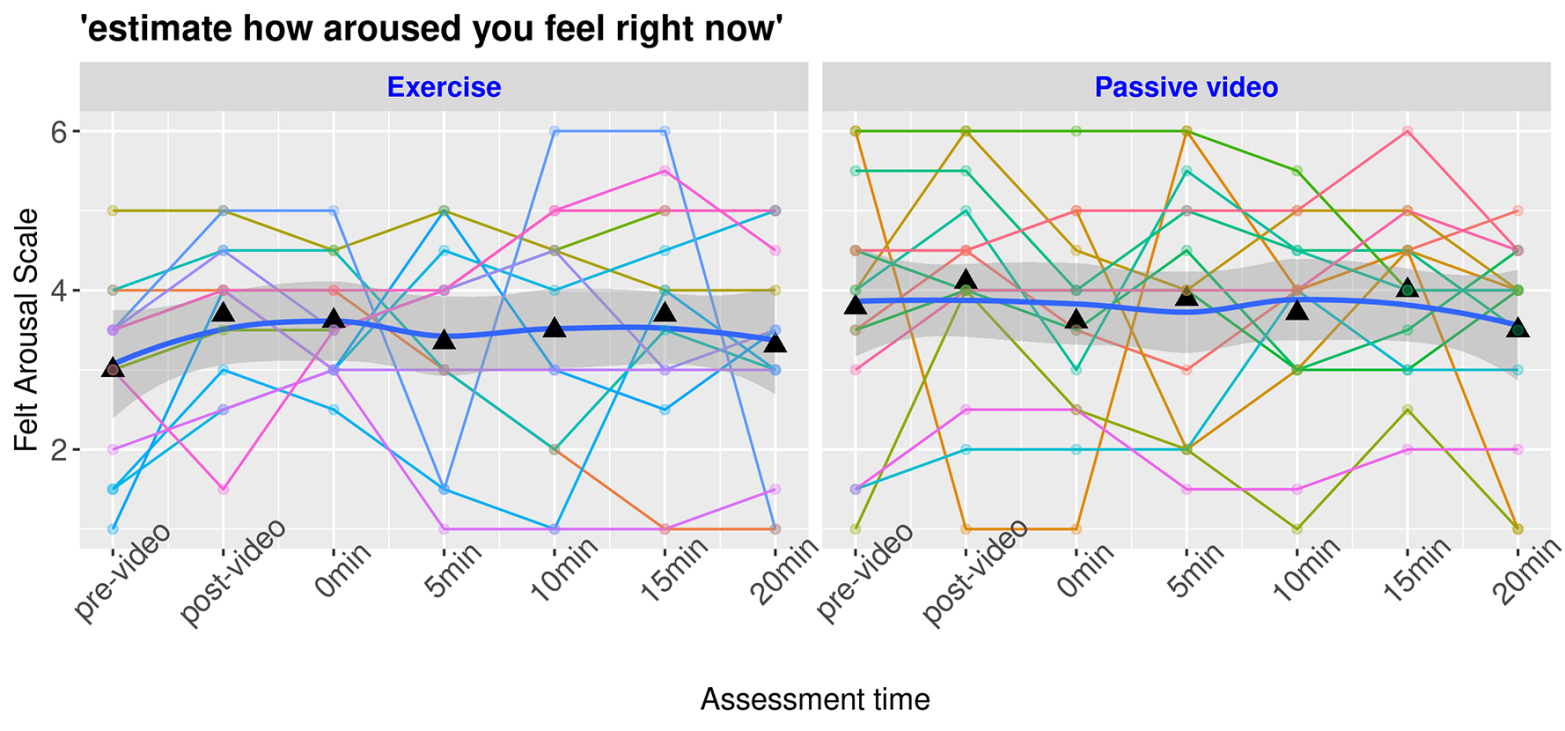
Protocol's Effect on the Felt Arousal Scale by Participants *Note.* Spaghetti plots with each curve representing a participant with a smooth representation of the group effect with confidence intervals of the curve. The bold blue line indicates the mean value of arousal surrounded by a darker gray area representing the confidence interval. Pre- and post video marks indicate evaluation before and after emotion induction. 0min through 20min marks indicate the time from the beginning of the condition (exercise or control).

The level of negative affect (*FS*) decreased in our sample during the 20 minutes of our protocol, *t*(106) = 2.79, *b* = .45, *d* = .54, *SE* = .16, *p* = .006. The post hoc power analysis revealed a power of 0.85. However, the PE session did not decrease negative affect more than the control condition over time, *t*(106) = -0.40, *b* = -.09, *SE* = .22, *d* = -.07, *p* = .70, as shown in Figure 4. The post hoc power analysis revealed a power of 0.07. The arousal (*FAS*) did not change over time, *t*(106) = -0.31, *b* = 0.04, *SE* = 0.15, *d* = 0.05, *p* = .80, and the PE and control groups were not significantly different, *t*(106) = 0.09, *b* = .02, *SE* = .21, *d* = .01, *p* = .92, as shown in Figure 5. The post hoc power analysis revealed a power of .08 and .05 respectively. However, some participants were observed almost sleeping while watching the control video. Each participant presented different patterns of FS and FAS and reacted differently in both groups (PE and control).

## Discussion

This pilot study is the first to our knowledge to examine the acute effects of PE on negative affect in adults with BPD. We hypothesized that PE would be safe, well accepted, and more effective than an emotionally neutral film in decreasing negative affect and arousal. Our findings show that PE is safe and well accepted, and participants in both conditions had a decrease in negative affect with a medium effect size, although the effect did not differ between the groups and arousal did not decrease during the protocol. However, the effects of PE on affect have been extensively studied and a meta-analysis shows its efficacy in increasing positive affect (Ekkekakis et al., 2011). The absence of a difference between the groups in our study is therefore unexpected. Moreover, we met many obstacles during this study that might explain the absence of group difference and therefore make it difficult to draw conclusion on our hypothesis.

One of the main reasons why our results were not significant was because this pilot study was underpowered to detect group effect. Indeed, the between-group analysis of affect valence difference had a power of .07 which is weak. According to the a priori analysis we made with the effect size we found, a sample size of 70 would have been sufficient to detect a significant group difference. However, because of the reasons detailed below, this effect size might be biased.

Despite the unanticipated findings, this experiment is useful and informative for future research investigating the acute effects of physical exercise on emotion regulation in BPD. First, no adverse effect was reported from the exercise sessions in this study, which indicates the safety of such an intervention. Second, every participant declared having appreciated the PE session with few negative feelings or discomfort toward it. However, this acceptability measure might not be the most valid and might be subject to biases. Third, the validated emotion induction procedure had unexpected effects. As reported by Chapman et al. (2010) and Kuo and Linehan (2009), it increased the mean level of negative affect in our sample. However, for nearly half of our sample, it had no effect or decreased the participants’ negative affect, as they either liked the thriller kind of movie or recognized the scene as being part of a movie they liked, suggesting that other mood induction content should be considered for future research of this nature. According to Rottenberg et al. (2018), non-response to mood induction is frequent and may affect the validity of a study. To avoid nonresponse, researchers might use multiple induction strategies at once, an instruction to strengthen the induction, or a longer induction. On the other hand, two participants reacted enough to the emotion induction such that they needed psychiatric care after the protocol. Those incidents indicate that this strategy might not be the safest available to induce negative affect in patients with BPD or that comorbid disorders (such as psychotic disorder) or previous traumas should be considered when selecting an induction strategy. Therefore, further research might attempt other induction strategies that better suit this population. For example, viewing negative emotional photos from the International Affective Picture Set paired with negative emotionally charged music (Lynn et al., 2012), reading emotionally charged sentences from the Velten validated battery (Velten, 1968), and/or vividly imagine personal negative situations (especially those relevant to BPD, such as abandonment experiences) triggered by a verbal script (Barnow et al., 2012). Finally, the neutral video that served as a control had a meditative effect on participants. Some participants were observed as almost sleeping while watching the video regardless of being probed every 5 minutes to rate their affect. Some participants also reported they meditated or used mindfulness strategies while looking at the video. Therefore, this control video might have had a meditating effect and effectively decreased the self-reported arousal level and increased the self-reported valence of affect. Indeed, meditation and mindfulness have been found to reduce negative affect (Goyal et al., 2014; Sathyanarayanan et al., 2019) and is currently used in Dialectic Behavioral Therapy (Linehan, 2014) to help reduce negative affect. Therefore, the control condition should not give participants the opportunity to use these techniques. For example, participants could be directed to do light stretching or articular warm-up for the same period as the PE session (Oberste et al., 2017). These results may be informative for researchers who are considering mood induction in experimental studies of PE in BPD.

Apart from the induction strategy and the control video, other factors might explain the absence of a difference between PE and the video in this study. The low physical activity level coupled with the high BMI of our sample might also be contributory. In a meta-analysis from Ekkekakis and colleagues (2011), inactive obese individuals were more likely to feel negative affect at low PE intensity than active individuals during a single bout of PE. Therefore, future research should investigate this effect in physically active individuals with BPD or with a BMI under 30.

Our findings resemble a previous investigation examining the effects of acute PE on core affect in adults with psychiatric illness (depressive disorder, bipolar disorder and anxiety disorder) using the FS and the FAS (Stanton et al., 2016). This study found a significant increase of valence only among participants with bipolar disorder or depressive disorder but not anxiety disorder. Furthermore, the PE session did not decrease the self-reported arousal level. Therefore, we can conclude that PE’s impact on affect likely differs depending on the specific psychiatric disorder. Emotion dysregulation is a component of all three of the disorders included in the Stanton et al. study, as well as BPD, with the latter associated with more severe emotion dysregulation than the other disorders (Gratz et al., 2016). Therefore, we can believe that PE might influence affect in BPD as well. Table 2 presents a set of potential solutions to overtake the main limitations encountered in our study to improve future studies.

**Table 2:** Study Limitations and Potential Improvements

Limitations	Suggestion
Heterogenous emotion induction (i.e., positive emotion following negative induction)	Three steps negative emotion induction (Kuo et al., 2014): Listening to emotionally charged music while watching emotionally charged photographs; Reading emotionally charged sentences; Vividly imagine personal negative emotion triggered by verbal script previously prepared.
Meditative effect of control condition	Use of placebo exercise (ex., light stretching, articular warm-up, Oberste et al., 2017)
Group discrepancy regarding household income and difficulties in emotion regulation	Recruit a larger sample to decrease group difference risk Or combined with a stratified randomization technique
Possible missed affect change after the ending of the measurement	Continue affect measurement for a period after the intervention (i.e., +5, +10, + 15 minutes)
Participants’ comorbid disorders were not reported	Accessing participants’ medical file to report comorbid disorders
Possible missed adverse effects	Adverse effects and safety should have been systematically assessed in the days following the investigation by calling participants directly
Possible invalid acceptability measure	Acceptability should have been measured using a validated questionnaire or a numerical scale to answer a single question to provide more information (Rabin et al., 2009).
Sample size	Based on a simulation analysis, a future well-powered study should include a total of 70 participants to reach a power of > 80% (Kumle et al., 2021)

On the other hand, this research has many strengths. The main strength is that it is the first study to include individuals with BPD to study the effect of PE. Also, the low to moderate PE intensity as self-selected by the participants optimizes PE benefits on affect (Ekkekakis et al., 2011). Moreover, we used core affect to assess physical activity effect on emotional feeling since it is known to be an effective way to characterize subjective feeling (Ekkekakis, 2013).

Future studies should use better suited negative emotion induction for adults with BPD (e.g., Velten validated battery). Other control strategies should also be used, such as light stretching or articular warm-up (LeBouthillier & Asmundson, 2015) considered as placebo PE. Watching a pleasant video at the end of the protocol could be used to improve participants’ affective valence before they complete the study, improving the safety of the protocol (Bernstein & McNally, 2017a, 2017b, 2018). Further work may study the impact of PE on affect in adults with BPD with ecological momentary assessments, which has been shown to be an efficient way to evaluate rapidly evolving phenomena in BPD (Santangelo et al., 2014). For example, the study of affect over a day after a PE session could elucidate the emotion regulation dynamics following PE. Other types, durations, and intensities of PE should also be tested, as these are all possible factors that might influence the affective response to PE (Ekkekakis et al., 2011). Finally, future exercise studies might evaluate the blood level of brain-derived neurotropic factor to measure the potential mediating role of this biomarker on affect in this population.

## Supplementary Materials

Data and analysis coding are available in open access in the Open Science Framework account of the first author (for access see Index of Supplementary Materials below).



St-AmourS.
CailholL.
RuoccoA. C.
BernardP.
 (2021). Supplementary materials to "Acute effect of physical exercise on negative affect in borderline personality disorder: A pilot study"
[Research data and analysis code]. PsychOpen. https://osf.io/ncd6r/
10.32872/cpe.7495PMC966741836397940

## Data Availability

For this article, a data set is freely available (St-Amour et al., 2021).
